# Pattern recognition: recent insights from Dectin-1

**DOI:** 10.1016/j.coi.2009.01.003

**Published:** 2009-02

**Authors:** Delyth M Reid, Neil AR Gow, Gordon D Brown

**Affiliations:** 1 Section of Immunology and Infection, Division of Applied Medicine, Institute of Medical Sciences, Foresterhill, University of Aberdeen, Aberdeen AB25 2ZD, United Kingdom; 2 Division of Immunology, CLS, Institute of Infectious Disease and Molecular Medicine, University of Cape Town, Cape Town, South Africa

## Abstract

The β-glucan receptor Dectin-1 is an archetypical non-toll-like pattern recognition receptor expressed predominantly by myeloid cells, which can induce its own intracellular signalling and can mediate a variety of cellular responses, such as cytokine production. Recent identification of the components of these signalling pathways, such as Syk kinase, CARD9 and Raf-1, has provided novel insights into the molecular mechanisms underlying Dectin-1 function. Furthermore, a broader appreciation of the cellular responses mediated by this receptor and the effects of interactions with other receptors, including the TLRs, have greatly furthered our understanding of innate immunity and how this drives the development of adaptive immunity, particularly Th17 responses. Recent studies have highlighted the importance of Dectin-1 in anti-fungal immunity, in both mice and humans, and have suggested a possible involvement of this receptor in the control of mycobacterial infections.

## Introduction

Higher animals are equipped with a limited array of germline encoded pattern recognition receptors (PRRs) that recognise highly conserved microbial structures known as pathogen associated molecular patterns (PAMPs). Engagement of PRRs by microbes or PAMPS directs appropriate innate immune responses and ultimately the development of specific adaptive immunity [1]. Although the toll-like receptor (TLR) family of PRRs makes crucial contributions to extracellular pathogen sensing and the development of immune responses, there has been a resurgence of interest in the non-TLR PRRs, of which the archetypical member is Dectin-1, the receptor for fungal β-glucans. The study of Dectin-1 has provided a number of new insights into the underlying mechanisms of innate immunity [2], and here we will review the most recent advances over the past two to three years. We will focus primarily on ‘trends’ of particular interest, including; Dectin-1 mediated signalling pathways, interactions of this receptor with other PRRs, directing adaptive immunity and the role of Dectin-1 in anti-microbial immunity.

## Dectin-1: a brief overview

**Figure 1:**
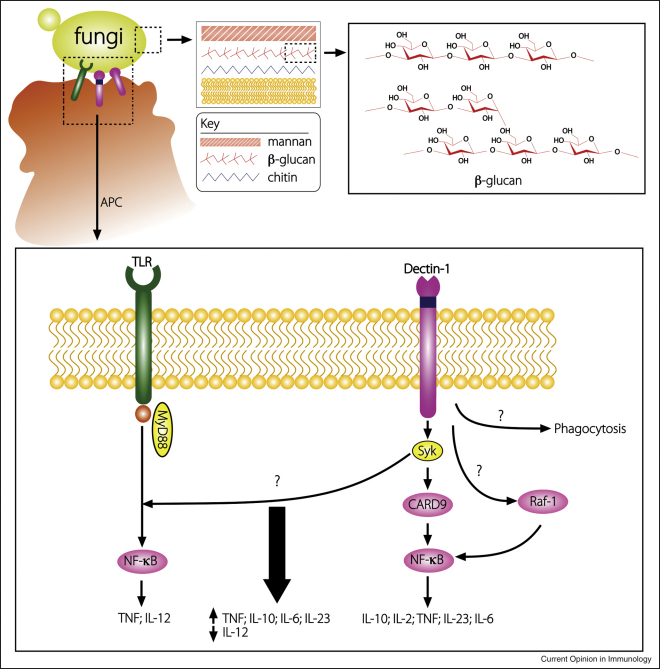
Schematic representation of the fungal cell wall and downstream cellular responses following engagement of Dectin-1 and TLRs on antigen presenting cells (APC). At the top, the interaction between the yeast cell of a fungus, such as *C. albicans,* is shown as well as the general architecture of the fungal cell wall, consisting of overlapping layers of highly mannosylated proteins, beta glucans (structure within the insert to the right) and chitin. Antigen presenting cells (APCs), including monocytes, macrophages and DCs, engage fungi and activate host responses via several PRRs including the Toll-like receptors (TLR) and Dectin-1. Dectin-1 is alternatively spliced into two functional isoforms, which differ by the presence or absence of a stalk region (shown in dark blue). Dectin-1 recognises linear or branched 1,3-linked β-glucan, which triggers intracellular signalling through at least two pathways, involving Syk kinase and Raf-1, inducing the production of several cytokines, including IL-10, TNF, IL-2, IL-6 and IL-23. TLRs, on the contrary, which recognise various mannosylated and other fungal cell wall structures, signal through the MyD88-Mal mediated NF-κB pathway and induce the production of both pro and anti-inflammatory cytokines, including TNF, IL-10, IL-12 and TGFβ. Co-stimulation of both receptors can amplify the production of cytokines, including TNF, IL-23, IL-10 and IL-6 while downregulating the production IL-12, influencing the resultant generation of adaptive immunity.

Dectin-1 is a glycosylated type II transmembrane receptor with a single extracellular C-type lectin-like domain (CTLD) and a cytoplasmic immunoreceptor tyrosine-based activation (ITAM)-like motif (also termed a hem-ITAM), and alternative splicing results in a number of isoforms, of which the two main functional ones are depicted in Figure 1. In both humans and mice, Dectin-1 is predominantly expressed on myeloid cells (monocytes/macrophage, dendritic cells and neutrophils) although limited expression has been reported on other cells, including various populations of lymphocytes [2].

Dectin-1 recognises β1,3-linked glucans, carbohydrates that are found in the cell walls of plants and fungi (Figure 1), and in some bacteria, and is the major receptor on myeloid cells for these molecules. Ligand binding is mediated by the CTLD and although the structure of this domain has been solved, and functionally examined by mutagenic analysis, the mechanism of carbohydrate recognition is still unclear. Dectin-1 also recognises ligand(s) on mycobacteria, and possibly some endogenous molecules, although the physiological roles of these interactions remain largely undefined [3].

Engagement of Dectin-1 on myeloid cells by β-glucans triggers a variety of cellular responses including DC maturation, ligand uptake by endocytosis and phagocytosis, the respiratory burst, the production of arachadonic acid metabolites and numerous cytokines and chemokines, such as TNF, CXCL2, IL-23, IL-6 and IL-10 [2]. However, the ability of Dectin-1 to trigger these responses directly is cell-type dependent, and may require, or be enhanced by, cooperative signalling from MyD88 coupled TLRs, as discussed below. In DCs, for example, Dectin-1 signalling can directly induce TNF production, yet in macrophages this response requires additional TLR co-stimulation [4,5^••^]. Although the underlying reasons are not fully understood, recent evidence suggests that the differences in cellular responses result, partly, from the actions of cytokines, such as GM-CSF and IFNγ [6^•^,7^•^]

## Recent trend 1: signalling pathways from Dectin-1

Dectin-1 was the first PRR outside of the TLR family that was found to be capable of inducing its own intracellular signals (Figure 1) [2]. Signalling from this receptor following ligand binding is mediated through the cytoplasmic ITAM-like motif that becomes phosphorylated by Src family kinases, providing a docking site for spleen tyrosine kinase (Syk). Despite involving both SH2 domains of Syk, only the membrane proximal tyrosine (Y_xxx_I/L_x7_*Y*_xx_L) of Dectin-1 was found to be required for signalling [8^••^,9]. This coupling was unexpected, and its discovery provided an important breakthrough in our understanding of activation signalling through single tyrosine-based motifs. Although the exact mechanism of signalling is still not fully understood, it has subsequently been shown to be utilised by at least two other receptors, CLEC9A and CLEC2 [10].

For Dectin-1, like most other myeloid expressed activation receptors, Syk is a pivotal kinase mediating many of the receptor's downstream cellular responses, such as cytokine production and induction of the respiratory burst [2]. While the components of the signalling pathway have yet to be fully elucidated, caspase recruitment domain 9 (CARD9), which assembles with BCL10 and MALT1, has been identified as an essential downstream adaptor linking Syk-coupled receptors to the canonical NF-κB pathway [11,12]. Dectin-1 can also induce the non-canonical NF-κB pathway, being the first PRR shown to do so [13^••^], and can activate NFAT, implicating these transcription factors in innate antimicrobial immunity, although the involvement of Syk in this response has not been established [14]. There is also evidence of Syk-dependent, but CARD9-independent, pathways, such as those leading to the induction of ERK, a MAP kinase regulating the Dectin-1-mediated production of cytokines, particularly IL-10 and IL-2 [15,16].

Dectin-1 can also induce intracellular signalling through Syk-independent pathways. Phagocytosis in macrophages, for example, does not require Syk, although this response still involves the ITAM-like motif of the receptor [2]. These pathways are still largely uncharacterised, but Dectin-1 was recently found to induce a Syk-independent pathway involving the serine–threonine kinase Raf-1 [13^••^]. This pathway was shown to integrate with the Syk pathway, at the level of NF-κB, and to be involved in controlling Dectin-1 mediated cytokine production.

## Recent trend 2: Interactions between Dectin-1 and other PRRs

The contact of intact pathogens with the host involves multiple PAMPs and their cognate PRRs, and it is the complex interaction of the responses mediated by these receptors, which directs the resultant innate response and ultimately the development of pathogen-specific immunity [17]. While our understanding of these receptor interactions is still in its infancy, the study of host–fungal interactions has arguably provided one of the best models in which to study these processes [18]. In addition to β-glucan, the fungal cell wall possesses other PAMPS (Figure 1) that are recognised by several PRRs, and cooperative recognition by these receptors is required for optimal innate anti-fungal responses [19^•^]. Intriguingly, recognition by some of these receptors was also found to occur at different stages during fungal uptake and phagosomal maturation [20^•^]. The location of PRRs in different intracellular compartments and the sequential sampling during microbial uptake, which has also been described for MyD88 versus TRIF signalling through TLR4 [21], may therefore be another means of tailoring innate responses to specific pathogens.

The interactions of Dectin-1 with other receptors, in particular, have provided some key new insights. In macrophages, cooperative signalling from both Dectin-1 and TLR-2 was shown to be required for the induction of TNF in response to fungi and was the first demonstration of such interactions between TLR and non-TLR receptors [2]. Although Dectin-1 displays a cell-type-specific ability to directly induce cytokine production, as described above, in both macrophages and DC, Dectin-1 can interact with other MyD88-coupled TLRs (TLR-2, TLR-4, TLR-5, TLR7, TLR-9), resulting in the synergistic induction of multiple cytokines including TNF, IL-10, IL-6 and IL-23 [4,22] (GD Brown *et al*., unpublished data). Interestingly, these interactions also result in the downregulation of IL-12 [23] (GD Brown *et al*., unpublished data), and the reciprocal regulation of IL-23 and IL-12 is likely to contribute to the development of Dectin-1 mediated Th17 responses (see below). How signalling from these receptors is integrated is unknown, but requires both the Syk and Raf-1 Dectin-1 mediated signalling pathways [4,13^••^], and may involve physical interactions at the plasma or phagosomal membrane [2,24].

In addition to the TLRs, Dectin-1 can also interact with several other plasma membrane proteins. In murine macrophages, Dectin-1 has been shown to cooperate with SIGNR1 for fungal-binding [25], and in human dendritic cells, the co-stimulation of DC-SIGN and Dectin-1 induces the metabolism of arachidonic acid [26]. Dectin-1 has also been shown to associate with the ubiquitous tetraspanin CD63 [27] as well as the immune cell-specific tetraspanin CD37, an interaction that is thought to stabilise Dectin-1 at the plasma membrane and be involved in regulating Dectin-1 mediated IL-6 production [28]. These interactions raise the possibility that Dectin-1 forms part of a tetraspanin mediated supramolecular signalling complex at the cell surface, which may involve several different PRRs.

## Recent trend 3: Dectin-1 and adaptive immunity

An important recent discovery is the ability of Dectin-1 to induce adaptive immunity. Stimulation of the Dectin-1 pathway in DC using highly purified β-glucans was shown to be able to induce the differentiation of Th17 and Th1 CD4^+^ T cells both *in vitro* and *in vivo*, and importantly these responses were independent of the TLR signalling pathways [5^••^]. Similar responses were also observed during fungal infections *in vivo*, and in humans [29]. Activation of DC with specific Dectin-1 agonists can drive the conversion of selected populations of Treg cells into IL-17 producing T cells [30], although Dectin-1/TLR costimulation may rather favour the development of Treg cells [16,31]. Why Dectin-1 actually promotes Th17 responses, in particular, is unclear, as these responses are thought to be detrimental during fungal infections [32], although this is still somewhat controversial [33,34]. However, Dectin-1 may ultimately prove to play a central role in setting the balance between pro-inflammatory and anti-inflammatory responses during fungal infections (see below).

Stimulation of Dectin-1 can also drive CD8^+^ T cell responses, and purified β-glucan was found to act as a potent adjuvant for CTL cross-priming *in vivo*, eliciting cytotoxic responses that could protect mice from experimental tumour challenge [35]. In addition, agonists of Dectin-1 could promote antibody responses *in vivo* [5^••^]. These data therefore show that stimulation via Dectin-1 can drive all arms of the adaptive immune response, suggesting that this receptor may be an appropriate immunotherapeutic target [36].

**Figure 2:**
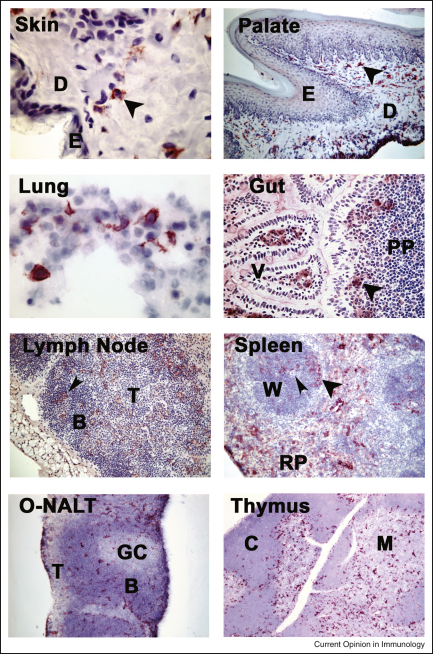
Dectin-1 expressing cells are positioned at portals of pathogen entry and sites of T cell development. Dectin-1 is expressed in the dermal layers of skin and palate (arrowheads) but not on the Langerhans cells. Dectin-1 is also expressed on lung APCs and in the gut, Dectin-1^+^ cells are located in the villi and Peyer's patches (arrowhead). In lymphoid tissues, Dectin-1^+^ cells are found in all areas including the germinal centres (small arrowhead in lymph node) and T cell regions around the central arteriole (small arrowhead) in the splenic white pulp. The organised nasal associated lymphoid tissues (O-NALT) demonstrate regional similarities to the lymph node. In the thymus, Dectin-1^+^ cells appear concentrated at the corticomedullary junctional regions. E: epidermis; D: dermis; PP: Peyers’ patch; B: B cell area; T: T cell area; GC: germinal centre; C: cortex; M: medulla.

The capacity of Dectin-1 to drive adaptive immune responses is thought to stem from its ability to induce DC maturation and the production of cytokines, such as IL-6 and IL-23 [5^••^]. However, these responses may also be influenced by the ability of the receptor to interact directly with lymphocytes. Dectin-1 was originally identified as a receptor for an unidentified endogenous ligand in T cells, and may function as a co-stimulatory molecule on APCs. Dectin-1 is also expressed in the medullary and corticomedullary regions of the thymus (Figure 2) and could have some influence on T cell development [2]; however, no abnormalities in T cell responses or cellular subsets have been observed in Dectin-1 deficient animals [37^•^,38^•^].

The responses mediated by Dectin-1 may also be involved in driving autoimmunity. Ligands of this receptor have been shown to induce autoimmune arthritis in SKG mice, and antibody-mediated inhibition of Dectin-1 could prevent the development of this disease [39^•^]. Similarly, blockage of Dectin-1 could prevent experimental autoimmune uveoretinitis, a Th1/Th17 disease induced by immunisation with retinal antigen in complete Freund's adjuvant (CFA) (Reid *et al*., unpublished), and it is likely that Dectin-1 will be implicated in other such autoimmune diseases in future. The development of these diseases may result, at least partly, from the ability of Dectin-1 to internalise and present endogenous antigens [40].

## Recent trend 4: role of Dectin-1 in anti-microbial immunity

**Figure 3:**
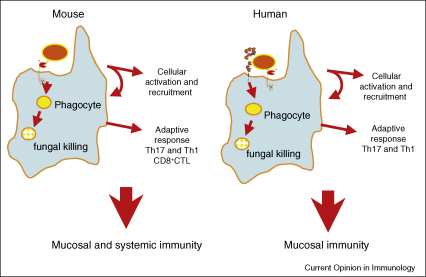
Diagrammatic representation of the role of Dectin-1 in anti-*Candida* immunity in mouse versus man. In mouse, sensing of *Candia albicans* by Dectin-1 on phagocytes (monocytes, macrophages, dendritic cells and neutrophils) results in the ingestion and killing of these organisms, and the induction of early inflammatory cytokines and chemokines. This results in cellular activation and recruitment of immune cells to the site of infection, and stimulates the development of adaptive immunity, including CD4^+^ and CD8^+^ T cell responses. In mice, defects in these responses, caused by deficiency of Dectin-1, result in susceptibility to systemic and mucosal candidiasis. In humans, Dectin-1 appears to function similarly; however, phagocytes deficient in Dectin-1 only show defects in cytokine responses, suggesting that other receptors, such as the mannose receptor, are responsible for fungal uptake and killing. The differences in the use of Dectin-1 presumably explain the susceptibility to mucocutaneous, but not systemic, candidiasis that is observed in humans with defects in this receptor.

β-Glucans are signature molecules for over a million fungal species and the identification of Dectin-1 as a receptor for these carbohydrates, and its expression in immune cells and at key portals of pathogen entry (Figure 2), immediately suggested a role in anti-fungal immunity. Indeed, Dectin-1 has been shown to mediate the recognition of several important fungal pathogens *in vitro*, including *Candida, Aspergillus, Pneumocystis* and *Coccidioides* species [3]. The exact role of Dectin-1 *in vivo* is still controversial, but there is strong evidence implicating this receptor in the control of infections with *C. albicans*, *P. carinii* and *A. fumigatus* [11,37^•^,38^•^,41]. These studies suggested that murine Dectin-1 is required for fungal uptake and killing and the induction of early inflammatory responses, data which clearly correlate with the *in vitro* functions described for this receptor (Figure 3). Also supporting a role for Dectin-1 is the ability of fungal pathogens to actively mask their β-glucan to avoid immune recognition, findings that have prompted interest in drugs that enhance the exposure of these carbohydrates [42–45].

A polymorphism in human Dectin-1 has been identified that is associated with an increased risk of mucocutaneous candidiasis (MG Netea *et al*., unpublished). Individuals homozygous for this polymorphism that generates an early stop codon in the CTLD of Dectin-1, lack expression of this receptor on the surface of their myeloid cells, and consequently these cells do not respond to β-glucans. Importantly these cells also show significant defects in the production of inflammatory cytokines in response to intact fungal organisms; however, the uptake and killing of these organisms is unaffected, indicating that alternative receptor pathways can mediate these activities. The functional differences to the murine system presumably explain the susceptibility of these individuals to mucocutaneous, but not systemic, fungal infections (Figure 3).

Studies from three independent laboratories have also implicated Dectin-1 in the recognition of mycobacteria. Interestingly, mycobacteria do not express β-glucans and ligand(s) interacting with Dectin-1 have not been identified. *In vitro* studies have suggested that Dectin-1, probably in collaboration with TLR2, is involved in the induction of cytokines, including IL-12, in response to these organisms and may play a role in mycobacterial phagocytosis [24,46,47].

## Summary and future directions

The study of Dectin-1 has provided substantial insights into the functioning of innate immunity and the development of adaptive immune responses. The underlying signalling pathways used by this receptor, and the effects of interactions with other receptors, are now starting to be unravelled, but there are still many gaps, particularly with regard to interactions between the Dectin-1 and the TLR pathways. Although accumulating data are pointing in the direction of Dectin-1 and its agonists as a potential way forward for adjuvant and immunotherapy development, further dissection of the complex relationships with other PRRs will facilitate improvements in current therapeutic and vaccination regimes.

Recent studies using receptor deficient mice and other murine models have demonstrated the importance of this receptor in anti-fungal immunity; studies that have been validated by the discovery of polymorphisms of human Dectin-1 that influence the susceptibility to fungal infections. Given the significant worldwide burden of candidiasis, more experimentation is required to better understand the role of Dectin-1 in these diseases. Similarly, the emerging role of Dectin-1 in anti-mycobacterial immunity and in autoimmunity warrants greater attention.

## References and recommended reading

Papers of particular interest, published within the period of review, have been highlighted as:

• of special interest
•• of outstanding interest
